# Co-modulated behavior and effects of differentially expressed miRNA in colorectal cancer

**DOI:** 10.1186/1471-2164-14-S5-S12

**Published:** 2013-10-16

**Authors:** Wei-Shone Chen, Ting-Wen Chen, Tzu-Hsien Yang, Ling-Yueh Hu, Hung-Wei Pan, Chung-Man Leung, Sung-Chou Li, Meng-Ru Ho, Chih-Wen Shu, Pei-Feng Liu, Shou-Yu Yu, Ya-Ting Tu, Wen-Chang Lin, Tony T Wu, Kuo-Wang Tsai

**Affiliations:** 1 Department of Surgery, Veterans General Hospital, Taipei, Taiwan; 2 Molecular Medicine Research Center, Chang Gung University, Taoyuan, Taiwan; 3 Bioinformatics Center, Chang Gung University, Taoyuan, Taiwan; 4 Department of Biotechnology and Laboratory Science in Medicine and Institute of Biotechnology in Medicine, National Yang Ming University, Taipei, Taiwan; 5 Institute of Biomedical Sciences, Academia Sinica, Taipei, Taiwan; 6 Department of Medical Education and Research, Kaohsiung Veterans General Hospital, Kaohsiung, Taiwan; 7 Department of Radiation Oncology, Kaohsiung Veterans General Hospital, Kaohsiung, Taiwan; 8 Clinical Genomics & Proteomics Core Laboratory, Department of medical research, Kaohsiung Chang Gung Memorial Hospital and Chang Gung University College of Medicine, Kaohsiung, Taiwan; 9 Genomics Research Center, Academia Sinica, Taipei, Taiwan; 10 Department of Surgery, Kaohsiung Veterans General Hospital, Kaohsiung, Taiwan; 11 School of Medicine, Yang-Ming University, Taipei, Taiwan

**Keywords:** microRNA, colorectal cancer, pathway enrichment analysis

## Abstract

**Background:**

MicroRNAs (miRNAs) are short noncoding RNAs (approximately 22 nucleotides in length) that play important roles in colorectal cancer (CRC) progression through silencing gene expression. **Numerous dysregulated **miRNAs simultaneously participate in the process of colon cancer development. However, the detailed mechanisms and biological functions of co-expressed miRNA in colorectal carcinogenesis have yet to be fully elucidated.

**Results:**

The objective of this study was to identify the dysfunctional miRNAs and their target mRNAs using a wet-lab experimental and dry-lab bioinformatics approach. The differentially expressed miRNA candidates were identified from 2 miRNA profiles, and were confirmed in CRC clinical samples using reported target genes of dysfunctional miRNAs to perform functional pathway enrichment analysis. Potential target gene candidates were predicted by an in silico search, and their expression levels between normal and colorectal tumor tissues were further analyzed using real-time polymerase chain reaction (RT-PCR).

We identified 5 miRNAs (miR-18a, miR-31, miR-96, miR-182, and miR-224) and 10 miRNAs (miR-1, miR-9, miR-10b, miR-133a, miR-143, miR-137, miR-147b, miR-196a/b, and miR-342) that were significantly upregulated and downregulated in colon tumors, respectively. Bioinformatics analysis showed that the known targets of these dysregulated miRNAs simultaneously participated in epithelial-to-mesenchymal transition (EMT), cell growth, cell adhesion, and cell cycles. In addition, we identified that several pivotal target gene candidates may be comodulated by dysfunctional miRNAs during colon cancer progression. Finally, 7 candidates were proven to be differentially expressed, and had an anti-correlationship with dysregulated miRNA in 48 CRC samples.

**Conclusion:**

Fifteen dysfunctional miRNAs were engaged in metastasis-associated pathways through comodulating 7 target genes, which were identified by using a multi-step approach. The roles of these candidate genes are worth further exploration in the progression of colon cancer, and could potentially be targets in future therapy.

## Background

Colorectal cancer (CRC) is one of the most common types of cancer in humans, and is the third leading cause of cancer-related death worldwide [1]. It is the consequence of a multi-step process caused by different genetic and epigenetic changes in numerous genes. MicroRNAs (miRNAs) are a class of non-protein coding RNA molecules of 18-25 nucleotides that exert their function on the base pairing between the seed region of miRNAs and the 3'-untranslated regions (3'-UTR) of target genes. Based on the degree of the complementary pairing between miRNAs and mRNAs, miRNAs either repress translation or promote the degradation of the mRNAs of target genes [2]. In the past decade, considerable evidence has shown that miRNAs are involved in the pathogenesis of human cancer, including CRC [2-7]. An miRNA profiling is an effective approach for high-throughput identification of dysregulated miRNAs during CRC progression, and numerous dysregulated miRNAs in colon cancer have been identified in previous studies [8-12].

The expression of tumor-suppressive miRNA usually suppresses tumor progression through silencing oncogene expression, and oncogenic miRNA frequently inhibits tumor-suppression gene expression, resulting in accelerating carcinogenesis. In colon cancer, most studies have focused on understanding the biological functions of several individual miRNAs, using both in vitro wet-lab experimental and in silico bioinformatics approaches, which provided invaluable information on miRNA-mRNA interactions. Even though miRNAs have been shown to target at genes involved in crucial steps within the protein interaction network [13]. It's been known that miRNAs usually repressed their target gene expressions slightly and alteration of an individual miRNA is insufficient to cause CRC. Therefore, in this study we are especially interested in the consequence of changes of a group of miRNAs in CRC. For those miRNAs that are changed systematically, the influence from a group of miRNAs is generally complex because of the regulation of an abundance of target genes. To further understand the detailed influences of miRNAs, the concept of miRNA regulatory modules (MRMs) was introduced [14-17]. MRMs are groups of miRNAs and their target genes that are thought to work together as a module, and that have correlated functions or are involved in similar biological processes. In 2005, Yoon and De Micheli suggested a method to identify MRMs that was based on miRNAs and their predicted target genes [16]. Since then, other methods have been proposed to identify modules from miRNA and mRNA expression profiles [14,15,17]. These studies have found that MRMs are highly enriched in known sets of biological pathways or GO biological process and shown that these MRMs may potentially serve as a model for understanding phenotype alterations or cancer pathogenesis.

In this study, we identified differentially expressed miRNAs in clinical samples of colon cancer, and then used the known targets for these miRNAs to accomplish the identification of potentially affected pathways. Differ from previous studies which used both miRNA and mRNA profiles to obtain a regulatory modules, we take advantage of pathway information to identified important target genes. Based on these pathways, we selected several potential candidate genes involved in colorectal progression. Our results provide biological evidence to support the hypothesis that miRNAs work as a team to regulate the expression of multiple targets, which leads to the alteration of cellular processes and specific biological pathways.

## Methods

### Clinical samples and RNA extraction

48-paired tumor and adjacent normal mucosa samples, and 8-paired metastatic liver tumor, primary tumor, and adjacent mucosa samples were obtained from CRC patients who underwent surgical operation at the Department of Surgery, Veterans General Hospital in Taipei, Taiwan. Informed consent was obtained from all patients. Among the 8 metastatic liver cancer patients, 2 patients were selected that exhibited metastatic liver tumors, primary tumors, and adjacent mucosa samples for ABI TaqMan low-density miRNA array analysis (Applied Biosystems, Foster City, CA, USA). The total RNA of the fresh tumor and non-tumor specimens was extracted using TRIzol reagent (Invitrogen, USA) according to the manufacturer instructions. In brief, tissue samples were homogenized in a 1 mL TRIzol reagent and mixed with 0.2 mL chloroform to extract protein, and the RNA was then precipitated using 0.5 mL isopropanol. The concentration, purity, and the amount of total RNA were determined using a NanoDrop 1000 spectrophotometer (NanoDrop Technologies Inc., USA).

### Stem-loop reverse transcription and real-time polymerase chain reaction

The primers were designed to detect mature miRNAs for stem-loop RT-PCR according to the methods described by Chen et al. [18]. One microgram of total RNA was reverse-transcribed using a stem-loop RT reaction with RT primers and SuperScript III Reverse Transcriptase according to the user manual (Invitrogen, Carlsbad, CA, USA). The reaction was performed under the following incubation conditions: 30 min at 16 °C, followed by 50 cycles of 20 °C for 30 s, 42 °C for 30 s, and 50 °C for 1 s. The enzyme was subsequently inactivated by incubation at 85 °C for 5 min. RT-PCR reactions were performed using an miRNA-specific forward primer and a universal reverse primer, and were conducted at 94 °C for 10 min, followed by 40 cycles of 94 °C for 15 s, and 60 °C for 32 s. Gene expression was detected using a SYBR Green I assay (Applied Biosystems, Foster City, CA, USA), and the expression levels of the miRNAs were normalized to that of U6. The expression levels of the predicted target genes were examined using RT-PCR analysis with a gene-specific primer, and were normalized to S26. The expression levels of the target genes were evaluated between the normal and cancer tissues using a paired *t *test. The difference was considered to be significant if the *P *value was less than 0.05. The individual primers used in this study are shown in Additional File 1.

### Pathway enrichment analysis

Known targets of the 15 dysregulated miRNAs were used to establish which biological processes or pathways these miRNAs might influence. A list of the known targets of each selected miRNA was identified using the MetaCore database. Both targets for upregulated miRNAs and targets for downregulated miRNAs were used for enrichment analysis using MetaCore. These statistically enriched pathways were further investigated to select candidate genes for further validation experiments.

### Selection of target genes for further investigation

After identifying the pathways potentially affected by the differentially expressed miRNAs, further validation was required to determine whether these reported targets were influenced in actual biological samples. Several enriched pathways relating to cancer development were targeted for study, such as cell proliferation, cell cycles, cytoskeleton remodeling, cell adhesion, EMT, and apoptosis. Within those pathways, the previously reported targets were selected for further examination. In addition to the reported miRNA targets, this study attempted to identify other potential targets. Potential miRNA targets were downloaded from TargetScan (Release 6.0: November 2011) [19]. Within the predicted targets, those with a context score greater than -0.2 were removed first because they were predicted to be low-efficacy targets [20,21]. After a list of high-efficacy targets was obtained, targets that were regulated by more than one differentially expressed miRNA were selected, except for genes targeted by both miR-196a and miR196b. Because miR-196a and miR-196b are in the same family and have the same group of predicted target genes, their target genes were treated as if they were targeted only once. Among those predicted high efficacy and co-regulated targets, 7 of them which were located at pathways that we previously selected, were noteworthy. These targets were further investigated to determine whether their expression patterns were consistent with the expected trends.

## Results

### Identifying miRNAs differentially expressed in colorectal carcinoma

Metastasis is the major cause of death in colon cancer patients. In this study, we attempted to identify dysfunctional miRNAs that are involved in CRC metastasis and progression. Therefore, we performed the expression profile of human miRNAs in primary cancer, its liver metastasis, and the corresponding normal mucosa of 2 CRC patients by using an ABI TaqMan low-density miRNA array approach. After completing the miRNA profiling of the 2 CRC samples, we identified the top 20 upregulated and top 20 downregulated miRNAs in tumor or liver metastases (Table 1). To further confirm these miRNAs, we examined the expression by using stem-loop RT-PCR in 6 additional CRC samples that contained primary tumors, and their corresponding liver metastases and mucosa. Finally, we identified 5 miRNAs (miR-18a, miR-31, miR-96, miR-182, and miR-224) and 10 miRNAs (miR-1, miR-9, miR-10b, miR-133a, miR-143, miR-137, miR-147b, miR-196a and 196b, and miR-342) that are frequently upregulated and downregulated in both colon tumors and liver metastasic tissue, respectively, compared with the adjacent normal and tumor areas (Additional File 2).

**Table 1:** The top 20 most differential up- and downregulated miRNAs are shown from 2 miRNA profiles.

Upregulated miRNAs	T1^a^	M1^b^	T2^c^	M2^d^
miR-183	25.6	22.9	3.8	9.1
**miR-31**	22.5	18.6	5.7 1	3.8
miR-135b	8.6	6.6	18.2	46.5
**miR-96**	8.3	8.6	6.7	65.3
**miR-224**	7.1	4.7	13.1	43.1
**miR-182**	5.2	5.7	2.1	6.5
miR-193a-5p	4.7	3.7	1.4	3.5
miR-409-5p	4.7	11.8	1	8.2
miR-18b	4.3	1.5	10.9	8.7
miR431	3.8	7.4	10.1	11
**miR-18a**	3.7	1.3	5.1	6.8
miR-221	3.2	1.1	4.8	5.3
miR-424	2.3	2	1.6	7.2
miR-25	1.6	1.9	3.8	5.1
miR-29b	1.5	2.6	2.4	7.3
miR-455-3p	1.5	2.2	9.7	46.2
miR-93	1.4	2.2	5.3	6.9
miR-483-5p	1.1	9.7	19.5	128
miR-455-5p	1.1	2.1	2.7	5.5
miR-122	1	201441.3	1	1370

**Downregulated miRNAs**				

**miR-137**	0.005	0.004	0.108	0.136
miR-133b	0.025	0.03	0.403	0.271
**miR-133a**	0.026	0.021	0.236	0.052
miR-138	0.038	0.061	0.566	0.255
miR-204	0.043	0.697	0.066	0.479
**miR-342-3p**	0.073	0.619	0.059	0.064
**miR-9**	0.079	0.223	0.291	0.447
miR-195	0.085	0.19	0.517	0.287
**miR-143**	0.097	0.118	0.435	0.366
miR-139-5p	0.112	2.1	0.167	0.87
miR-140-3p	0.113	0.269	0.343	0.554
**miR-196a/b**	0.125	0.001	0.031	0.015
**miR-1**	0.146	0.012	0.586	0.384
**miR-10b**	0.156	0.037	0.779	0.279
miR-218	0.166	0.297	0.213	1.2
let-7b	0.203	0.506	0.726	0.646
miR-149	0.204	0.162	0.174	0.801
miR-145	0.205	0.153	0.438	0.514
**miR-147b**	0.251	0.152	0.122	0.025
miR-215	0.489	0.23	0.318	0.619

The expression patterns of 15 miRNA candidates that proved consistent with the profile data after further confirmation in addition to the 6 CRC samples are shown in bold.

**a. **Primary colon cancer: adjacent mucosa from patient 1

**b. **Liver metastases: adjacent mucosa from patient 1

**c. **Primary colon cancer: adjacent mucosa from patient 2

**d. **Liver metastases: adjacent mucosa from patient 2

### Dysregulated miRNA coexpression in colon cancer

**Figure 1:**
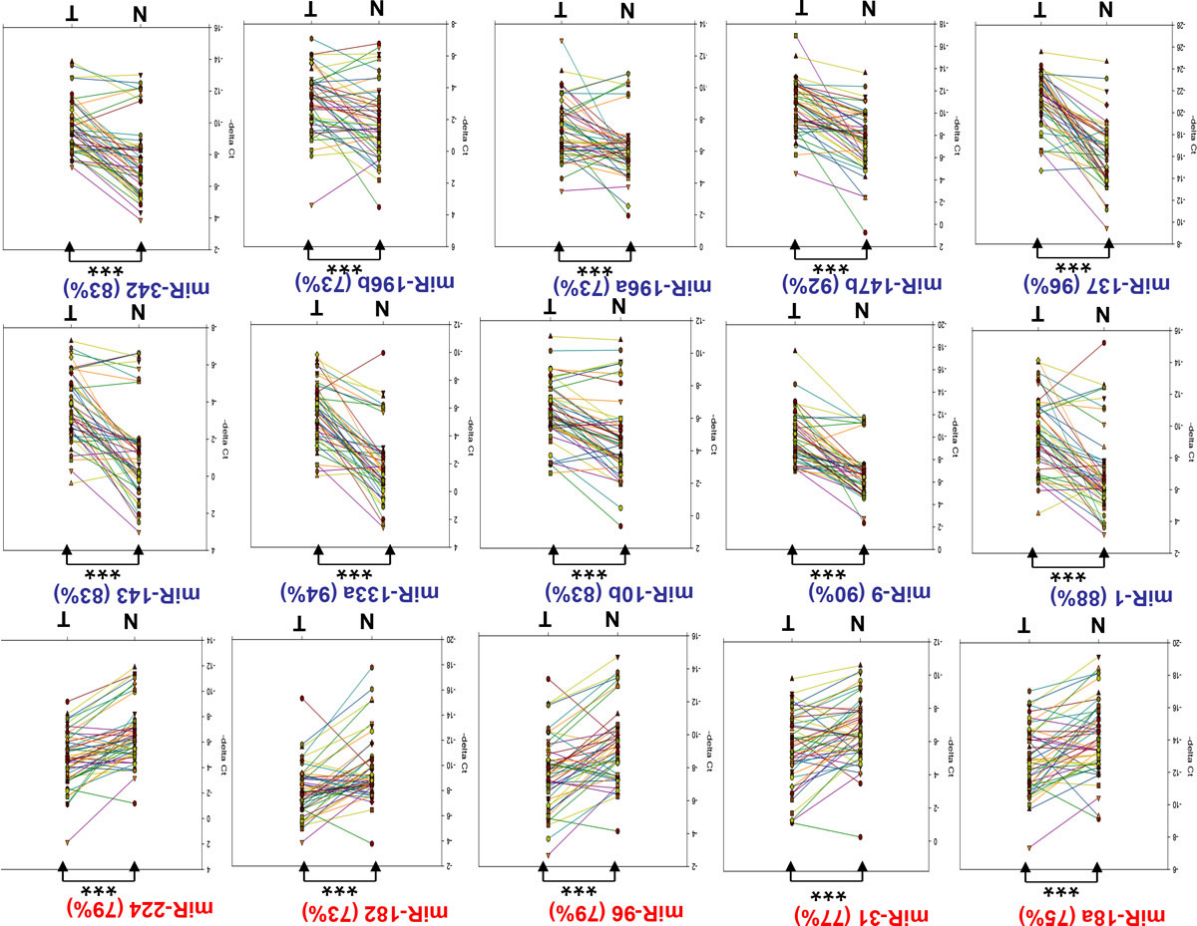
**Expression levels of 15 dysregulated miRNAs in 48 CRC patients**. Expression of 15 miRNAs in the CRC tissues from 48 patients was analyzed using quantitative stem-loop PCR with U6 as an internal control. The percentage of upregulation or downregulation of individual miRNA in the 48 CRC patients is shown at the top of each panel. All samples were assessed in triplicate and analyzed using paired *t *tests (*P *< 0.05 was considered significant; NS: not significant, * *P *< 0.05, ** *P *< 0.01, *** *P *< 0.001).

We further examined the expression level of miRNA candidates in 48 CRC samples by using the stem-loop RT-PCR approach. The expression levels of miR-18a, miR-31, miR-96, miR-182, and miR-224 were significantly upregulated in at least 70% of the CRC samples (*P *< 0.0001). In other groups, we found that miR-1, miR-9, miR-10b, miR-133a, miR-137, miR-143, miR-147b, miR-196a/b, and miR-342 were significantly downregulated in at least 70% of the samples (*P *< 0.0001) (Figure 1). These results indicated that these miRNA candidates may play a pivotal role in colon cancer progression. By reviewing previous studies, we learned that most of these miRNAs have been reported as differentially expressed miRNA in colon cancer [5,22-24]. However, a comprehensive understanding of the biological functions of these miRNAs in CRC is lacking, particularly for miR-10b, miR-96, miR-133a, miR-147b, miR-196a/b, and miR-342. We further observed that the 5 upregulated miRNAs simultaneously exhibited upregulation in 62% of the CRC samples (30 out of 48) and the 10 downregulated miRNAs were simultaneously silenced in 58% of the CRC samples (28 out of 48). Our data implies that these miRNAs tend to dysregulate simultaneously in CRC, and these miRNAs may regulate cancer-associated signaling pathways by comodulating a group of critical genes in CRC.

### Biological pathways analysis

**Figure 2:**
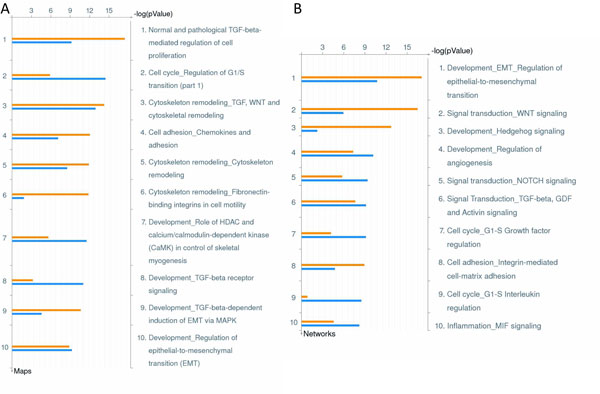
**Pathway and process enrichment results of reported target genes of 15 dysregulated miRNAs**. (A) The respective pathway enrichment results and (B) the process enrichment results of known targets for both upregulated miRNA (orange) and downregulated miRNA (blue) were generated by MetaCore. The horizontal axis is the negative log transformation of the *P *value, which indicates the probability that these processes (or pathways) are raised by chance. In pathway enrichment, numerous pathways related to cancer, such as cell proliferation, cell cycles, cytoskeleton remodeling, and EMT, are statistically enriched. A similar outcome is found in process enrichment, and many cancer- or metastasis-correlated processes are enriched.

Pathway enrichment analysis enables us to comprehensively investigate the association between a group of miRNAs and cancer progression by using computational methods [6,25,26]. However, the high false-positive rates of these prediction programs result in low reliability. To more precisely determine what specific biological pathways are regulated by these differentially expressed miRNAs, we used known target genes to perform pathway enrichment analysis in this study. First, we downloaded 366 and 741 reported known targets for these upregulated and downregulated miRNAs respectively from the MetaCore database that have high-quality, manually-curated miRNA and miRNA target information. We further performed pathway enrichment statistical tests on these known target gene sets. MetaCore calculates probability according to a hypergeometric distribution formula, and provides the final *P-*value estimations of the likelihood of a particular pathway being enriched and selected at random. For a list of interested genes, if the number of interested genes found in one pathway is significantly higher than expected, then that particular pathway is, by definition, enriched and is reported as such. Pathway enrichment analysis provides us with clues regarding which biological functions or specific pathways these known targets may contribute to on a cellular level. The top 10 enriched pathways in Figure 2A show that most of these enriched pathways are related to cancer development or metastasis, such as cell proliferation, cell cycles, cytoskeleton remodeling, cell adhesion, or EMT. Regarding the enriched processes (Figure 2B), many are also related to EMT regulation, cell growth, angiogenesis, cell adhesion, cell cycles, and so on. Our results indicated that the targets of the differentially expressed miRNAs are predominantly involved in the development and metastasis of CRC.

### Locating potential target genes of dysregulated miRNAs in colon cancer

Because miRNAs could act as either oncomiR or tumor-suppressor miRs depending on their target genes, we selected several known and predicted target genes to further examine whether their expression levels were changed in colon cancer samples after statistical pathways enrichment analysis. Numerous target prediction tools for miRNAs have been developed, such as PITA, miRanda, PicTar, and miRBase Targets [27-30]. From them, we used the TargetScan tool, which provides target predictions through the conserved complementarily to the seed region of miRNAs. Although TargetScan is shown to have the best performance [31], a high false-positive rate is still a problem because each miRNA has up to several hundred targets. Therefore, reasonably narrowing down the target genes enables us to more efficiently locate the potential target genes that participate in colon cancer progression. In this study, we applied 2 concepts to locate the potential target genes: (1) Co-expressed miRNAs are believed to share similar biological functions and participate in the same signaling pathways in CRC; and (2) co-expressed miRNAs may comodulate the same critical gene, which results in efficiently blocking or promoting the signaling pathway progression. Therefore, we selected candidate target genes that conformed to these 2 concepts. We considered both predicted and reported data, and obtained a list of candidates, as shown in Table 2.

**Table 2:** Target genes of 15 dysregulated miRNA were selected for RT-PCR examination in this study

Target type	Group	Gene Symbol	miRNA	Expression level in colon cancer^#^
**predicted**	**Group I**	GDNF	miR133a, miR9	Down**
	**miRNA ↓ **	MYH9	miR133a, miR9	Up*
		RNF111	miR1, miR9	Down*
	
	**GroupII**	GNA13	miR182, miR96	Down*
	**miRNA ↑ **	HBEGF	miR182, miR96	Down***
		LAMC1	miR182, miR96	Up***
		PPP2R3A	miR182, miR96	Down***

**known**	**Group III**	Bim	miR10b	NS
	**miRNA ↓ **	CDC42	miR1	NS
		collagen I	miR143	Up***
		CyclinD1	miR1	Up**
		PDGFRB	miR9	NS
		PPP2CA	miR133	NS
		Versican	miR9	Up**
		calmodulin	miR196a, miR143	NS
	
	**Group IV**	ATM	miR18a	NS
	**miRNA ↑ **	FBXW7	miR182	NS
		FZD3	miR31, miR182	NS
		SMAD2	miR18a	Up**
	
	**Group V**	CREB1	miR10b, miR182	Up***
		FBXW11	miR133, miR96, miR182	NS
	**Mixed**	IGF1	miR1, miR18a	Down***
		MAP3K14	miR137, miR31	NS
		MSN	miR133a, miR96	Up***
		PDGFRA	miR342, miR182	NS
		SMAD4	miR1, miR224	NS
		RASA1	miR1, miR96, miR182, miR31	NS

# Paired *t *test results: NS: not significant, * *P *< 0.05, ** *P *<0.01, *** *P *< 0.001

For known targets, we selected the 6 most reported targets in the enriched pathways, excluding those that have no predicted target site in TargetScan or were predicted to target only controversial dysregulated miRNAs; that is, we selected 3 groups of known targets. The first 2 target groups have been reported and predicted as only downregulated miRNAs (Group III) or as only upregulated miRNAs (Group IV). The third group of targets reportedly targets either up- or downregulated miRNA and is predicted by both upregulated and downregulated miRNAs (Group V). For these predicted targets, we only considered the targets which were predicted to be targeted by more than one of our selected differentially expressed miRNAs to increase our accuracy. From the predicted targets downloaded from TargetScan, there are 179 predicted targets co-regulated by at least 2 upregulated miRNAs and 172 predicted targes co-regulated by at least 2 downregulated miRNAs. Among those predicted co-regulated targets, we further selected 7 that are involved in the enriched pathways we previously derived from known targets. Within these 7 predicted targets, 3 are potential targets for downregulated miRNAs (Group I), and 4 are potential targets for upregulated miRNAs (Group II). Based on the concepts of co-function and co-modulation, we reasonably reduced our candidate list, which was composed of both reported and predicted potential target genes (Table 2).

### Examination of the expression levels of miRNA targets

**Figure 3:**
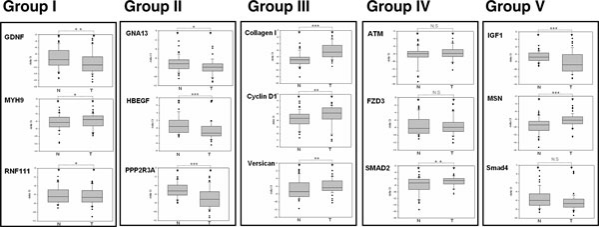
**Expression levels of target gene candidates of dysregulated miRNAs in 48 CRC patients**. Expression of target genes in the CRC tissues of 48 patients was analyzed using quantitative stem-loop PCR with S26 as an internal control. All samples were assessed in triplicate and analyzed using paired *t *tests (*P *< 0.05 was considered significant; NS: not significant, * *P *< 0.05, ** *P *< 0.01, *** *P *< 0.001).

Table 2 presents 27 pivotal genes (20 known and 7 predicted target genes), which may be co-regulated by dysregulated miRNA, that may contribute to the impairment of several biological functions (EMT regulation, cell growth, cell adhesion, and cell cycles) during colorectal progression. We further examined the expression levels of these target genes in 48 CRC samples by using the RT-PCR approach. As shown in Figure 3 and Table 2, 14 out of the 27 candidate genes showed significant differential expression (including 8 upregulation and 6 downregulation), and 13 out of the 27 gene transcript levels demonstrated no significant difference in the CRC tissues compared to the adjacent normal tissues. In general, when miRNA regulated its target genes by directly destroying the mRNA, the miRNA expression level should be negatively correlated with the targeted gene expression in the cells. Therefore, the target genes should have an increased expression level in the tumor samples, and the target genes should have been identified from the downregulated miRNAs sets (Groups I and III). Conversely, target genes targeted by upregulated miRNAs sets should have decreased expression levels (Groups II and IV). According to our data, 7 out of the 27 candidate genes' expression levels were observed to be negatively correlated with their individual miRNAs regulators. Among them, MYH9 was shown to provide an oncogenesis role in cancer cell migration [32,33]. Our data indicates that MYH9 may be a putative novel target gene of miR-133a and miR-9, and the upregulated expression level of MYH9 may be caused by the low expression of miR-133a and miR-9 in CRC (Figure 3, Group I).

We also identified 3 cancer-associated genes, GAN13, HBEGF, and PPP2R3A, with significantly decreasing expression levels in colon cancer (Figure 3, Group II). They were selected from predicted target genes of miR-182 and miR-96 in Group II. Among them, PPP2R3A was implicated as a tumor-suppressor gene contributing to cell transformation [34]. However, we do not have a complete understanding of the regulation mechanism of PPP2R3A and its relationship with miRNAs in CRC. In this study, we provided a finding that miR-182 and miR-96 may contribute to silence PPP2R3A expression in CRC. In Group III, we identified 3 putative candidates, Collagen I, Cyclin D1, and Versican, which were significantly upregulated in CRC tissues. Although previous studies have shown that miR-196a directly targeted Collagen I, and miR-143 repressed the expression of Cyclin D1 and Versican [35-39], the regulation mechanism of Collagen I, Cyclin D1, and Versican by miRNAs in CRC is not understood. In addition, we have also identified novel miRNAs candidates, miR-143, miR-1, and miR-9, which have the potential to regulate Collagen I, Cyclin D1, and Versican, respectively (Table 2). In our data, we have observed conflicting results in the expression pattern between the miRNAs and anticipated target genes. These conflicting results may be because one gene is usually regulated by a complex mechanism. In addition, previous systematic genome-wide studies have indicated that the regulation effects of miRNAs may be detectable at the protein-synthesis level, although no significant changes may exist at the mRNA expression level [31,40].

## Discussion

MiRNAs play either a tumor-suppressive or an oncogenic role depending on their target genes, and may modulate cancer growth, cell cycles, and migration in CRC [5,22,23,41]. In general, multiple miRNAs are simultaneously dysfunctional during carcinogenesis. One miRNA regulates the expression of more than one target, and one gene can be regulated by more than one miRNA. This type of many-to-many relationship complicates the study of relevance between miRNAs and their targets, and results in difficulties in comprehensively investigating the altered expression of a group of miRNAs. It has been proposed that miRNAs work together to regulate their target genes in the regulatory network, which differs from previous MRM identification methods [14-17] that considered the sequence similarity or expression profiles, but not biological pathways. These MRMs are likely to simultaneously involve several biological pathways. MRMs identified in a previous study were shown to be enriched in biological pathways, and that these MRMs are likely to be functionally correlated [17]. In addition, regarding pathways, the predicted targets for differentially expressed miRNAs in cancer cells show a broad range of changes, which provide clues to explain abnormal phenotypical alterations [42]. Together with these studies, our results suggest that, beginning with pathway analysis, one may successfully integrate several crucial targets of dysregulated miRNAs to provide an explanation of cancer development.

Compared with previous studies, most of dysfunctional miRNAs detected in this study had been reported in colorectal cancer [5,22-24]. However, the biological functions of these miRNAs in CRC remained unclear, particularly for miR-10b, miR-96, miR-133a, miR-147b, miR-196a/b, and miR-342. In this study, a comodulated concept was adopted to reasonably identify the miRNA targets. To locate the modules of these differentially expressed miRNAs that may participate in colon cancer; we constructed miRNA and mRNA relationships by using direct targets. We focused on direct targets because a previous study suggested that the high through-put of mRNA and miRNA expression profiles did not yield more accurate prediction outcomes of the protein product levels [43] compared to the direct targets. In order to obtain more reliable miRNA-mRNA relationships, we used reported targets instead of predicted targets that were used in previous studies [14,15,17,44]. Accordingly, these targets should improve the accuracy of potentially altered pathways. Numerous metastasis- and cancer-related pathways were enriched. Our results also support the theory of MRMs that these miRNAs target at many targets and work as a module, which leads to abnormalities in cancer development or metastases.

Our approach toward potential miRNA target gene selection, namely the use of co-regulation, the same pathway, and the context score, may also provide biologically reasonable rules for novel miRNA target prediction fields. Although not all of our predicted targets were supported through qPCR, the potential targets selected through these criteria are still convincing. For example, when we prepared this manuscript, Rasheed et al. reported that miR-182 can silence GAN13 protein expression, but cannot alter mRNA levels by targeting its 3'-UTR, which results in inhibiting prostate cancer cell migration and invasion [45]. Their results support our findings in which GNA13 is predicted as a putative novel target of miR-182, and its transcriptional levels have a slight negative correlation with miR-182 expression in clinical samples (Table 2). Although the detailed functions of miR-182 silencing the GNA13 expression in colon cancer requires further investigation, their data helps support the reliability of our strategy of novel target selection.

Through further experimental validation, we learned that certain target mRNA expression levels changed in an unexpected manner. This inconsistency has also been observed in a recent study, in which the expression levels of miRNAs and genes in the same MRM were associated by up to 69% [17]. Although we only retained reported targets and high-efficacy and coregulated predicted targets, several of these exhibited nonsignificant or conflicting expression patterns. One gene is usually regulated by numerous regulatory factors in actual biological systems, and the regulatory effects of miRNAs may be observed at either the mRNA expression level or the protein synthesis level [31,40]. For example, based merely on miRNA regulation, we expected the expression level of GDNF should be upregulated, but some other factors have already been shown to involve in regulation of GDNF expression in cancers. DNA hypermethylation contributes to the low expression of GDNF in several human cancers [46-48]. Satio et al. also found that the promoter region of GDNF was more highly methylated in active inflamed mucosa than in quiescent mucosa in ulcerative colitis patients [49]. These results could address why GDNF significantly reduced its expression in CRC compared to the adjacent normal tissue. Here, we only focused on investigating the mRNA expression levels of targeted genes because of the scarcity of clinical samples, and budgetary limitations. Although the mRNA expression levels of the targeted genes did not completely fit our expectations because they were under the regulation of the miRNAs, these expression levels coupled with the expression-level changes of the miRNA from the clinical samples are invaluable.

## Conclusion

Fifteen dysregulated miRNAs were identified by screening clinical samples. We have successfully examined the major biological functions and signaling pathways of these co-expressed miRNAs, and have provided an approach to reasonably narrow down the target genes of co-expressed miRNAs in colon cancer. Both dysfunctional miRNAs and their comodulating target genes are worth further exploration in the progression of colon cancer, and could potentially be targets in future therapy.

## Competing interests

The authors declare that they have no competing interests.

## Authors' contributions

TWC executed this study and prepared the draft of the manuscript. YTT and SYY were responsible for RT-PCR validation. SCL and MRH performed pathway enrichment analysis. THY, LYH, PFL, CWS and HWP assisted with the tissue preparation and RNA extraction. WSC, WCL, TTW and KWT supervised the study and edited the manuscript.

## Supplementary Material

Additional File 1 Sequences of primers for miRNA and target gene detection Click here for file

Additional File 2 **Expression levels of dysregulated miRNAs in 6 liver metastasis patients**. Expression of miRNAs was examined in primary tumors, metastatic liver tumors, and the corresponding normal mucosa of 6 CRC patients using RT-PCR with U6 as an internal control. Click here for file

## Declarations

Publication of this article was funded by National Science Council, Taiwan (NSC 102-2320-B-010-029).

This article has been published as part of *BMC Genomics *Volume 14 Supplement 5, 2013: Twelfth International Conference on Bioinformatics (InCoB2013): Computational biology. The full contents of the supplement are available online at http://www.biomedcentral.com/bmcgenomics/supplements/14/S5.
