# Supplementary material for: Co-modulated behavior and effects of differentially expressed miRNA in colorectal cancer
Source: BMC Genomics. 2013 Oct 16;14(Suppl 5):S12. doi: 10.1186/1471-2164-14-S5-S12 (PMC3852113; doi:10.1186/1471-2164-14-S5-S12)
Supplement: Additional File 1 — Sequences of primers for miRNA and target gene detection [file 1471-2164-14-S5-S12-S1.pdf]

Additional file 1. primer sequence list	
microRNA RT primer	
Primer	sequence 5' to 3'
miR-18a-RT	CTCAACTGGTGTCGTGGAGTCGGCAATTCAGTTGAGCTATCTGC
miR-31-RT	CTCAACTGGTGTCGTGGAGTCGGCAATTCAGTTGAGAGCTATGC
miR-96-RT	CTCAACTGGTGTCGTGGAGTCGGCAATTCAGTTGAGAGCAAAAA
miR-182-RT	CTCAACTGGTGTCGTGGAGTCGGCAATTCAGTTGAGAGTGTGAG
miR-224-RT	CTCAACTGGTGTCGTGGAGTCGGCAATTCAGTTGAGAACGGAAC
miR-1-RT	CTCAACTGGTGTCGTGGAGTCGGCAATTCAGTTGAGATACATAC
miR-9-RT	CTCAACTGGTGTCGTGGAGTCGGCAATTCAGTTGAGTCATACAG
miR-10b-RT	CTCAACTGGTGTCGTGGAGTCGGCAATTCAGTTGAGCACAAATT
miR-133a-RT	CTCAACTGGTGTCGTGGAGTCGGCAATTCAGTTGAGCAGCTGGT
miR-137-RT	CTCAACTGGTGTCGTGGAGTCGGCAATTCAGTTGAGCTACGCGT
miR-143-RT	CTCAACTGGTGTCGTGGAGTCGGCAATTCAGTTGAGGAGCTACA
miR-147b-RT	CTCAACTGGTGTCGTGGAGTCGGCAATTCAGTTGAGTAGCAGAA
miR-196a/b-RT	CTCAACTGGTGTCGTGGAGTCGGCAATTCAGTTGAGCCCAACAA
miR-342-3p-RT	CTCAACTGGTGTCGTGGAGTCGGCAATTCAGTTGAGACGGGTGC
microRNA GSF primer	
Primer	sequence 5' to 3'
miR-18a-GSF	CGGCGGTAAGGTGCATCTAGTG
miR-31-GSF	CGGCGGAGGCAAGATGCTGGCA
miR-96-GSF	CGGCGGTTTGGCACTAGCACAT
miR-182-GSF	CGGCGGTTTGGCAATGGTAGAA
miR-224-GSF	CGGCGGCAAGTCACTAGTGGTT
miR-1-GSF	CGGCGGTGGAATGTAAAGAAGT
miR-9-GSF	CGGCGGTCTTTGGTTATCTAGC
miR-10b-GSF	CGGCGGTACCCTGTAGAACCGA
miR-133a-GSF	CGGCGGTTTGGTCCCCTTCAAC
miR-137-GSF	CGGCGGTTATTGCTTAAGAATA
miR-143-GSF	CGGCGGTGAGATGAAGCACTGT
miR-147b-GSF	CGGCGGGTGTGCGGAAATGCTT
miR-196a-GSF	CGGCGGTAGGTAGTTTCATGTT
miR-196b-GSF	CGGCGGTAGGTAGTTTCCTGTT
miR-342-3p-GSF	CGGCGGTCTCACACAGAAATCG
Universal-R	CTGGTGTCGTGGAGTCGGCAATTC
U6-F	CTCGCTTCGGCAGCACA
U6-R	AACGCTTCACGAATTTGCGT
Target genes real-time PCR primers	
Primer	sequence 5' to 3'
GDNF-F	GTCACTGACTTGGGTCTGGG
GDNF-R	GCCTGCCCTACTTTGTCACT
MYH9-F	AAGCTGGTATGGGTGCCTTC
MYH9-R	CTTGGGCGGGTTCATCTTCT
RNF111-F	GAGGCAACACCTAGGGACAC
RNF111-R	GTCTCCGTAACGAACTGCCA

GNA13-F	TGCCTGTCTCGGGAAAAGAC
GNA13-R	ACGTTGCTGTAGATGGTGGG
HBEGF-F	AGTCCGTGACTTGCAAGAGG
HBEGF-R	CCTCTTCTTCCCTAGCCCCT
LAMC1-F	TGGGCATTCTTCTGTCTGTACAA
LAMC1-R	GCCACCCATCCTCATCAATC
PPP2R3A-F	CCTCTTGCACATCCCTGTGT
PPP2R3A-R	AGGCCTGTTGGGGATAGTCT
Bim-F	AGGCAATCACGGAGGTGAAG
Bim-R	TGGAAGCCATTGCACTGAGA
CDC42-F	CGTGACCTGAAGGCTGTCAA
CDC42-R	ACACACCTGCGGCTCTTCTT
collagen I-F	GCCAAGACGAAGACATCCCA
collagen I-R	GGCAGTTCTTGGTCTCGTCA
CyclinD1-F	CGCCCCACCCCTCCAG
CyclinD1-R	CCGCCCAGACCCTCAGACT
PDGFRB-F	GGTGACACTGCACGAGAAGA
PDGFRB-R	GTCCCCAATGGTGGTTTTGC
PPP2CA-F	TGGAGATGTGCATGGGCAAT
PPP2CA-R	TGGTGATGCGTTCACGGTAA
Versican-F	GACTACTGTCCTTGTGGCCC
Versican-R	CGGTAAAGACCCGCATCACT
Calmodulin-F	CACTGGGTCAGAACCCAACA
Calmodulin-R	AAGACTCGGAATGCCTCACG
ATM-F	ACAAGCCTCCAGGCAGAAAA
ATM-R	TGTTGCTACAATCAGCTCCGT
FBXW7-F	CCCAGCAAGGACAGTTGGAA
FBXW7-R	GAGGAGAGTTGGTGAACGGG
FZD3-F	CAATGGAGCCATTCCACCCT
FZD3-R	AGGCCAAGGAACACCAAACA
SMAD2-F	GCTGGCCTGATCTTCACAGT
SMAD2-R	CCAGAGGCGGAAGTTCTGTT
CREB1-F	AGACTTCAGCACCTGCCATC
CREB1-R	TGTCCATCAGTGGTCTGTGC
FBXW11-F	ATGGCAGCGAGTGATCTCAG
FBXW11-R	ATTTGGAGGGCCATCTGTGG
IGF1-F	TTTCAACAAGCCCACAGGGT
IGF1-R	TTGAGGGGTGCGCAATACAT
MAP3K14-F	CCCATGCTACAGAGGGCAAA
MAP3K14-R	ATGAGCCAGGGACTTTGAGC
MSN-F	GTGACCACCATGGATGCAGA
MSN-R	AAAGAACAGGCGCTGAGTGA
PDGFRA-F	GACGGTCTTGGAAGTGAGCA
PDGFRA-R	GTACCACCCCCTCACTGTTG
SMAD4-F	GGAGGTGGCCTGATCTTCAC
SMAD4-R	CTTGGTGGATGCTGGATGGT

RASA1-F	CCAAACTGCCCACTTCGTTG
RASA1-R	CTGGTTAGTTGGAGGAGCGG
S26-F	CCGTGCCTCCAAGATGACAAAG
S26-R	GTTCGGTCCTTGCGGGCTTCAC
