# Supplementary figures and images for: Co-modulated behavior and effects of differentially expressed miRNA in colorectal cancer
Source: BMC Genomics. 2013 Oct 16;14(Suppl 5):S12. doi: 10.1186/1471-2164-14-S5-S12 (PMC3852113; doi:10.1186/1471-2164-14-S5-S12)

**miR-18a**  
(4/6)

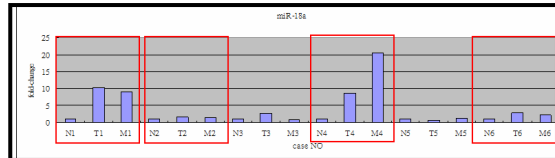

**miR-31**  
(5/6)

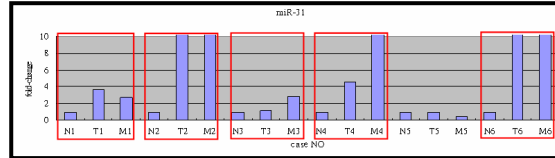

**miR-96**  
(5/6)

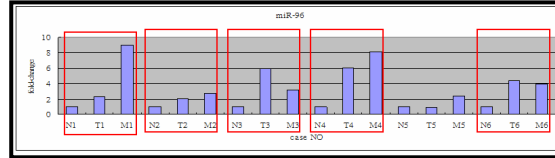

**miR-182**  
(6/6)

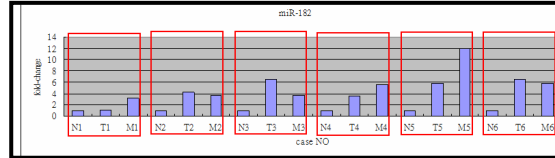

**miR-224**  
(5/6)

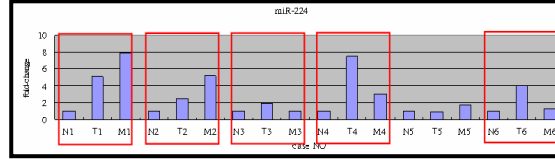

**miR-1**  
(6/6)

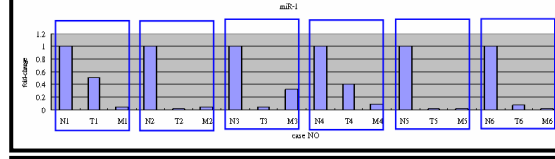

**miR-9**  
(5/6)

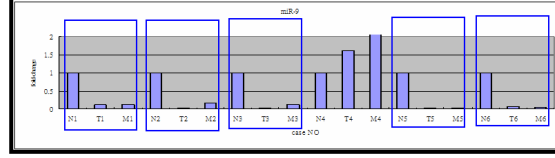

**miR-10b**  
(5/6)

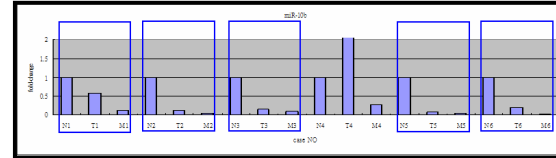

**miR-133a**  
(6/6)

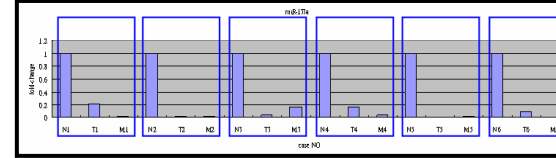

**miR-137**  
(5/6)

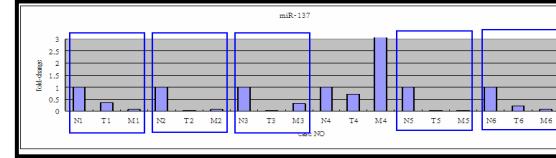

**miR-143**  
(6/6)

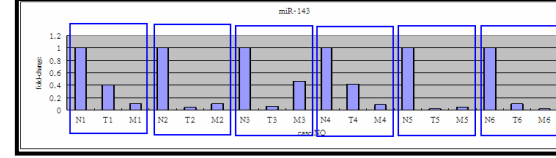

**miR-147b**  
(5/6)

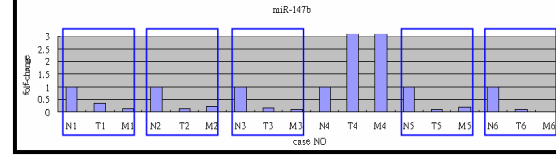

**miR-196a/b**  
(5/6)

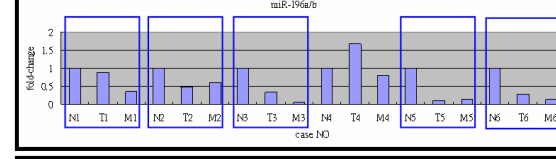

**miR-342**  
(6/6)

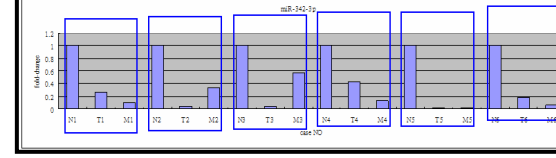

Supplement: Additional File 2 — Expression levels of dysregulated miRNAs in 6 liver metastasis patients. Expression of miRNAs was examined in primary tumors, metastatic liver tumors, and the corresponding normal mucosa of 6 CRC patients using RT-PCR with U6 as an internal control. [file 1471-2164-14-S5-S12-S2.pdf]
